# Improved Thermal Infrared Image Super-Resolution Reconstruction Method Base on Multimodal Sensor Fusion

**DOI:** 10.3390/e25060914

**Published:** 2023-06-09

**Authors:** Yichun Jiang, Yunqing Liu, Weida Zhan, Depeng Zhu

**Affiliations:** 1 The College of Electronic and Information Engineering, Changchun University of Science and Technology, Changchun 130022, China; jiangyichun@mails.cust.edu.cn (Y.J.); zhanweida@cust.edu.cn (W.Z.); zhudepeng@mails.cust.edu.cn (D.Z.); 2 National Demonstration Center for Experimental Electrical, Changchun University of Science and Technology, Changchun 130022, China

**Keywords:** thermal infrared imaging, super-resolution reconstruction, multimodal sensors, information fusion

## Abstract

When traditional super-resolution reconstruction methods are applied to infrared thermal images, they often ignore the problem of poor image quality caused by the imaging mechanism, which makes it difficult to obtain high-quality reconstruction results even with the training of simulated degraded inverse processes. To address these issues, we proposed a thermal infrared image super-resolution reconstruction method based on multimodal sensor fusion, aiming to enhance the resolution of thermal infrared images and rely on multimodal sensor information to reconstruct high-frequency details in the images, thereby overcoming the limitations of imaging mechanisms. First, we designed a novel super-resolution reconstruction network, which consisted of primary feature encoding, super-resolution reconstruction, and high-frequency detail fusion subnetwork, to enhance the resolution of thermal infrared images and rely on multimodal sensor information to reconstruct high-frequency details in the images, thereby overcoming limitations of imaging mechanisms. We designed hierarchical dilated distillation modules and a cross-attention transformation module to extract and transmit image features, enhancing the network’s ability to express complex patterns. Then, we proposed a hybrid loss function to guide the network in extracting salient features from thermal infrared images and reference images while maintaining accurate thermal information. Finally, we proposed a learning strategy to ensure the high-quality super-resolution reconstruction performance of the network, even in the absence of reference images. Extensive experimental results show that the proposed method exhibits superior reconstruction image quality compared to other contrastive methods, demonstrating its effectiveness.

## 1. Introduction

Thermal infrared imaging is a passive imaging technology that detects the thermal radiation passively emitted by objects to form an image [1]. It has the advantages of strong anti-interference ability and the capability to distinguish between targets and backgrounds. Therefore, super-resolution reconstruction (SR) has been widely applied in fields such as remote sensing imaging [2,3,4], target tracking [5,6,7], and autonomous driving [8,9], etc. However, compared with visible light imaging, infrared imaging equipment usually has limited spatial resolution, resulting in lower imaging quality. Therefore, to overcome this limitation, super-resolution reconstruction technology has become an important research field. The super-resolution technology can restore high-frequency information from low-resolution images, which can improve the resolution of infrared images and enrich image details.

Currently, due to the continuous improvement of computational device performance and the increasing maturity of deep learning technology, deep learning-based SR methods have become the mainstream solution to SR problems. Compared with interpolation-based [10], reconstruction-based [11], and sparse representation-based methods [12], they have significant performance advantages. The focus of the single image super-resolution reconstruction (SISR) network is mainly on the reasonable allocation of network resources to high- and low-frequency information reconstruction. SISR relies on the mapping relationship between high- and low-resolution information (HR&LR) images solidified in the weight parameters through training and does not introduce effective external information.

Compared with collecting infrared images, high-quality visible light images are more easily obtained and possess higher spatial resolution. Although they operate in different spectral bands, a significant amount of complementary information exists, making it feasible and effective to guide infrared image super-resolution using complementary information from visible light images. Some research has made progress, but several key issues remain:

(1) In the case of multimodal super-resolution, the large resolution difference between infrared and visible images leads to a significant decrease in the accuracy of reconstructed infrared images. High-performance super-resolution reconstruction networks, especially their feature extraction and information transformation mechanisms, still require further research.

(2) Due to the imaging mechanism of thermal infrared sensors, the quality of infrared images remains poor despite high pixel resolution. Existing methods that use simulated degradation and train their inverse process are limited by the quality of the infrared images used as labels, making it difficult to effectively enhance high-frequency details in infrared images.

(3) The existing multimodal super-resolution reconstruction methods have not fully considered the cases where the reference image is missing or of poor quality, which leads to a sharp degradation in the performance of the network and poor quality of the reconstructed images.

To address these issues, we proposed a thermal infrared image super-resolution reconstruction method based on multimodal sensor fusion. The method consists of a novel neural network architecture, a new hybrid loss function, and corresponding training strategies. The input infrared image is reconstructed through the network, during which multimodal features are continuously extracted and fused to obtain a high-quality, high-resolution thermal infrared image. The proposed loss function is used to constrain the network to ensure that the thermal infrared information in the image is not erroneously altered. Moreover, the proposed training strategy ensures that the network can still correctly reconstruct thermal infrared images even when the reference images are missing or of poor quality.

Our main contributions are as follows:

(1) We proposed a super-resolution reconstruction network that continuously fuses information from different scales of visible light images in the iterative process to reconstruct low-frequency and high-frequency information in infrared images, solving the problem of accuracy decline caused by large resolution difference between infrared and visible light images.

(2) We proposed a hierarchical dilated distillation module that can adaptively extract features of different scales, with strong representation ability and fewer learnable parameters.

(3) We proposed an information transformation module based on attention mechanism, which calculates pixel-level correlation between infrared and visible light features to reduce the interference of redundant and unrelated information on reconstructed images, improve information fusion efficiency, and suppress the blurring phenomenon in the infrared image reconstruction process.

(4) We designed a hybrid loss function for multimodal super-resolution to supervise the network to obtain more high-frequency features from visible light images and ensure the style of infrared images does not deviate by adversarial loss, retaining richer details and more thermal infrared information.

(5) We proposed a modal switching training strategy to solve the problem of degraded performance in reference-based super-resolution reconstruction of thermal infrared images when the reference image is missing, improving the network’s robustness.

## 2. Related work

### 2.1. Image Super-Resolution Reconstruction Based on Neural Networks

As an ill-posed problem, super-resolution reconstruction is limited in its reconstruction and generalization capabilities if relying solely on manually designed prior methods. As a result, neural networks, which are powerful implicit function fitters, have been employed due to their effectiveness in fitting complex mappings in image processing. Since the introduction of the first convolutional neural network for image super-resolution, SRCNN [13], the use of neural networks in this field has grown exponentially, with a primary focus on optimizing network structures. Early research works such as VDSR [14], SRResNet [15], and EDSR [16] have significantly improved network feature expression ability and reconstruction quality by deepening the network and incorporating the residual structure concept. However, increasing network depth and width to a certain extent becomes inefficient, resulting in diminishing performance gains. To further enhance reconstruction quality and efficiency, new model structures have been designed specifically for SR tasks, optimizing reconstruction while maintaining low complexity. These improvements include multi-scale feature extraction [17,18], feature reuse [19,20,21], and attention mechanisms [22,23]. Such modifications introduce prior knowledge into the network structure, enhancing the model’s adaptability to SR tasks while reducing the network’s dependence on learnable parameters and training data.

In addition to improving network structure, efforts have been made to better train neural networks to generate realistic and detailed texture details. References [24,25] investigated several commonly-used loss functions in image restoration and provided guidance for loss function design in super-resolution reconstruction. Although these loss functions calculate the difference between predicted and real data, they may produce significant blur or aliasing artifacts due to the diversity of mappings. Consequently, the use of generative adversarial learning is being explored to obtain the implicit distribution of real images from the dataset [13,26,27], guiding the network to generate clearer reconstruction results. However, this technique often results in apparent reconstruction errors that are difficult to avoid. Despite the current advancements in network structure, loss functions, and training methods for SR, there remains substantial room for further improvement.

### 2.2. Multimodal Reference-Based Super-Resolution Reconstruction

Compared to SISR, reference-based SR is a technique that uses additional guiding images to transfer relevant structural information to the target image in order to achieve high-quality super-resolution reconstruction [28,29]. In early research, multimodal reference-based super-resolution (multimodal SR) reconstruction mainly used filtering-based [30,31], optimization-based [32], and sparse representation-based [33] methods. However, these methods faced difficulties in reconstructing HR images, especially when there were large differences in image structure or resolution between modalities. Currently, the main method used is learning-based. By utilizing the powerful fitting ability of deep learning, texture conversion and transmission between modalities can be achieved.

However, in recent research, impressive reconstruction quality has been achieved by studying the correlation between the source image and the reference image. Despite this progress, these methods still adhere to the traditional SR training method, which simulates the downsampling process and then learns its inverse process, producing images similar to the original collected data [34,35,36]. The method suffers from the limitations of the low resolution and imaging mechanism of the thermal infrared sensor. Compared to reconstructing high-quality visible light or near-infrared images, it is difficult to reconstruct high-quality infrared images using this method and many texture details may be lost.

In order to solve this problem, some studies have designed fusion strategies to synthesize visible light and infrared image information, and introduce visible light texture while performing SR of infrared images [37]. However, although this method supplements some details, the generated image not only produces incorrect texture but also does not conform to the thermal information distribution in the infrared source image due to its imperfect network structure, loss function, and supervision design. Therefore, further research and improvement are still needed to develop effective fusion strategies that can better preserve the thermal information distribution and generate high-quality HR images. Additionally, the use of appropriate evaluation metrics is essential to ensure that the generated images meet the requirements of practical applications.

## 3. Proposed Method

Thermal infrared radiation can be affected by various factors when reaching imaging sensors, such as motion blur, optical blur, and electronic noise, leading to degradation in the quality of infrared images. Super-resolution reconstruction techniques for thermal infrared images are often considered the inverse process to address these issues. However, the pixel size of thermal infrared sensors is larger, and diffraction and scattering effects are more pronounced. As a result, even when the resolution is the same, thermal infrared images appear blurrier. Traditional super-resolution reconstruction methods obtain HR and LR infrared image pairs through simulated downsampling and training the SR mapping in reverse is not effective in reconstructing ideal HR infrared images. We believe that preserving the original infrared thermal information is necessary, while predicting some high-frequency information reasonably can make the reconstructed image more visually appealing. Therefore, our research focused not only on restoring the information in the original infrared image but also on using visible light images to guide neural networks to predict and reconstruct high-frequency information in thermal infrared images to improve the overall quality of reconstructed images. To achieve our goal, we designed specific network structures, loss functions, and training strategies.

### 3.1. Network Architecture

**Figure 1:**
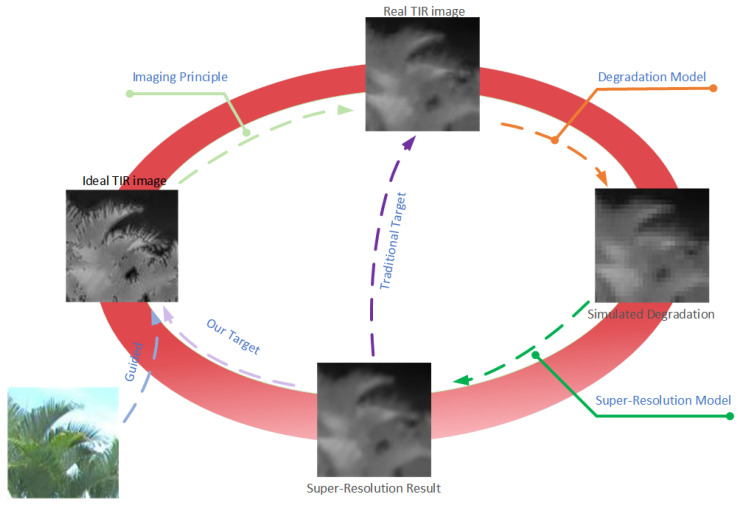
The target of our method.

The network structure is shown in Figure 1. Our proposed network consists of three parts: the primary feature encoding subnetwork, the super-resolution reconstruction subnetwork, and the high-frequency detail fusion subnetwork. Subsequently, we will explicate the operational principles, design concepts, and particular implementations of each component.

#### 3.1.1. Primary Feature Encoding Subnetwork

The Primary Feature Encoding Subnetwork is used to map the input image to a feature space for further processing. It primarily consists of an infrared feature encoder and multiple visible light feature encoders. The infrared feature encoder is only used before the first stage of super-resolution reconstruction subnetwork to encode the input infrared image $I_{LR}^{TIR}$ into primary feature $f_{b}^{TIR}$ using a straightforward convolutional layer, which can be represented by the following equation:

(1) $$\begin{array}{c} f_{b,1}^{TIR}=\sigma \left(W_{enc}^{TIR}*I_{LR}^{TIR}+B_{enc}\right)\\ f_{b,n}^{TIR}=f_{o,n-1}^{}\left(n=2,3\cdots N\right) \end{array}$$

where $W_{enc}^{TIR}$ represents the filter for encoding thermal infrared images, $B_{enc}$ represents the bias value, $f_{o,n}^{}$ represents the output feature map for the n-th stage of super-resolution reconstruction subnetwork, σ(x)=max(x,0) represents the rectified linear unit, and ∗ represents the convolution operation. The visible light feature encoder uses multiple convolutional layers with varying specifications, depending on the super-resolution reconstruction multipliers, to encode visible light images $I_{}^{VIS}$ at different scales. These layers construct a feature pyramid to generate visible light image features $f_{b,n}^{VIS}\left(n=1,2,\cdots ,N\right)$ corresponding to the various stages of the reconstruction process. Mathematically, the process can be expressed as follows:

(2) $$f_{b,n}^{VIS}=\sigma \left(W_{n}^{VIS}*I^{VIS}+B\right)\left(n=1,2\cdots N\right)$$

where $W_{n}^{VIS}$ represents the encoding filter for visible light images used in the n-th stage and *N* represents the total number of stages. Unlike the infrared encoding filter, the visible light encoding filter is used in each stage, and the corresponding filter uses different convolution kernel sizes and strides. Specifically, for the filter $W_{b,n}^{VIS}$ in the n-th stage, the stride is set to $2^{N-(n-1)}$ to output the current infrared feature map size, and the convolution kernel size is designed as $2^{N-(n-1)}+1$ to prevent the loss of pixel information in the image. By using this method, we can mainly introduce the low-frequency features of visible light images in the early stage of reconstruction, and focus more on the high-frequency details of visible light images in the later stage of reconstruction and fusion of details.

#### 3.1.2. Super-Resolution Reconstruction Subnetwork

**Figure 2:**
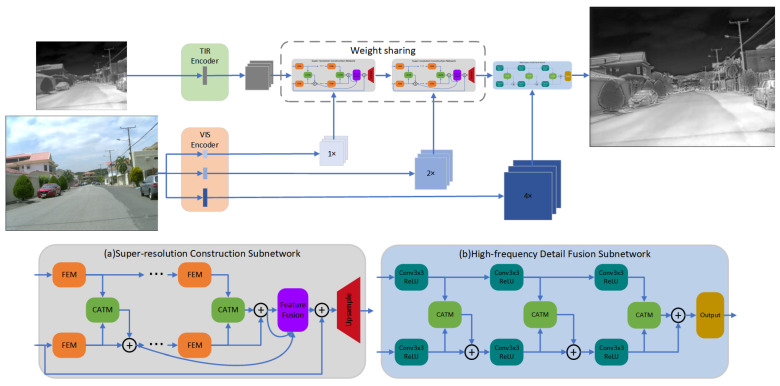
The architecture of our proposed network.

As shown in Figure 2a, the Super-Resolution Reconstruction Subnetwork is the core component of our network, which aims to restore and enhance the resolution and texture details of thermal infrared images. Note that for different stages of super-resolution (SR), we use the same subnetwork for super-resolution reconstruction, with shared weights and identical structure. For this subnetwork, we designed the Feature Extraction Module (FEM), Cross-Attention Transformation Module (CATM), and Upsampling Module (UM) for efficient extraction of structural information from different modal images. By measuring the degree of correlation between multimodal images, we achieved effective texture transfer and super-resolution reconstruction.

**Figure 3:**
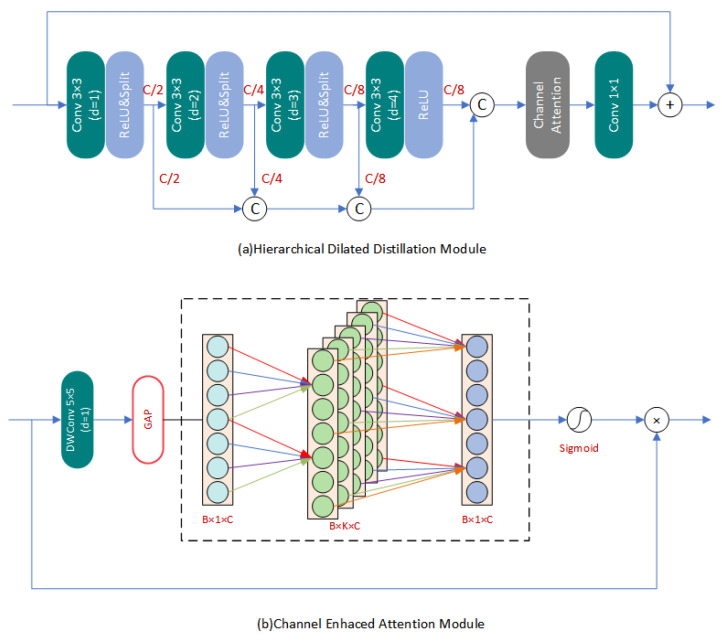
The basic unit of Feature Extraction Module.

**Figure 4:**
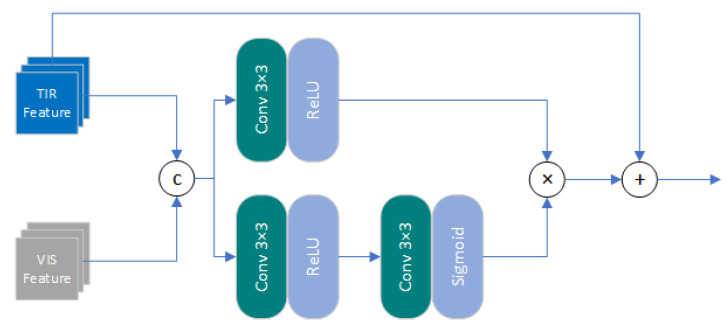
Cross-Attention Transformation Module.

- **Feature Extraction Module (FEM):** We employed the same structure for the FEM used to process both infrared images and visible light features. This approach is based on the fact that visible light features have been previously adjusted to a feature space that matches the infrared features during the primary feature encoding process. Batch normalization layers in the network can destroy the original contrast of images in image reconstruction tasks according to some existing research. Therefore, we specifically removed all batch normalization layers in the network to improve reconstruction performance, reduce redundancy operations, and increase training and inference speed. Our FEM consisted of a series of improved Hierarchical Dilated Distillation Modules (HDDM) as presented in Figure 3a. We designed a multi-scale distillation fusion mechanism for visible or infrared input feature maps $f_{in}^{FEM}$, which sequentially passes through filters with different dilation rates to separate different frequency components of different image features. This process enhances the representational capacity of the network. After each filter output, the feature map is split into two equal parts along the channel dimension. One part $f_{s1}^{FEM}$ is directly passed on to the subsequent steps for feature fusion, while the other part $f_{s2}^{FEM}$ continues to extract features. This operation can be represented as
  (3) $$\begin{array}{c} f_{MS,1}^{FEM}=\sigma \left(W_{MS,1}*f_{in}^{FEM}+B_{MS,1}\right)\\ \left[f_{s1,n-1}^{FEM},f_{s2,n-1}^{FEM}\right]=f_{MS,n-1}^{FEM}\\ f_{MS,n}^{FEM}=\sigma \left(W_{MS,n}*f_{n-1}^{FEM}+B_{MS,n}\right),n=\left(2,3,4\right) \end{array}$$
  where $W_{MS,n}^{FEM}$ represents the n-th filter in HDDM. Then, we concatenated all the $f_{s1}^{FEM}$ in HDDM into one vector for subsequent operations. This operation can be represented as
  (4) $$\begin{array}{c} f_{c}^{FEM}=F_{cat}\left(f_{s1,1}^{FEM},f_{s1,2}^{FEM},\cdots ,f_{s1,M-1}^{FEM},f_{MS,M}^{FEM}\right)\\ f_{a}^{FEM}=F_{ca}\left(f_{c}^{FEM}\right) \end{array}$$
  where $F_{ca}\left(\cdot \right)$ represents our improved Channel Enhanced Attention Module (CEAM), as shown in Figure 3b. Firstly, in low-level visual tasks such as super-resolution, it is more important to focus on the image structure information. Directly using global average pooling to extract information is not appropriate. Therefore, we first introduced a depthwise separable convolution at the front end of CEAM to process the features of each channel. Then we performed the operation of global average pooling.
  We also utilized a 1-D convolution to process the compressed channel information, inspired by previous literature [38]. This approach reduces the computational and parameter complexity. We not only avoided the dimensionality reduction operation in channel attention, but also elevated the channel dimension to form multiple subspaces for different aspects of information. By combining different dimension features, we achieved more flexible information interaction between channels.
  Inspired by previous research, such as EDSR, we introduced local residual learning into the feature extraction module. This approach can effectively alleviate the potential problem of gradient disappearance in the parameter optimization process, making it possible to construct a deeper super-resolution reconstruction network. To perform point-wise add operation between the output feature map and the input feature map, we set a filter with a convolution kernel size of 1 × 1 at the end. This filter fuses the multi-scale information previously extracted and matches the number of channels with the input feature map. This operation can be represented as follows:
  (5) $$f_{out}^{FEM}=\sigma \left(W_{out}^{FEM}*f_{2}^{FEM}+B_{out}^{FEM}\right)+f_{in}^{FEM}$$
- **Cross-Attention Transformation Module (CATM):** In order to guide the process of infrared image SR with visible features, we constructed a Cross-Attention Transformation Module to obtain the attention map of relevant information from the input visible light features and transfer the useful information. The structure of the CATM are shown in Figure 4.
  Given the input of infrared and visible feature maps $f_{in}^{TIR}$ and $f_{in}^{VIS}$, which are obtained by the feature extraction module processing the primary features of infrared and visible light, respectively. After $f_{in}^{TIR}$ and $f_{in}^{VIS}$ were concatenated into a tensor $f_{in}^{CATM}$, they were input into the attention branch. Unlike previous attention mechanisms, we did not limit the estimation of attention maps to channel or spatial dimensions, but constructed a pixel-level attention mechanism. Firstly, $f_{in}^{CATM}$ was filtered by a 3 × 3 convolutional kernel to extract effective features in the feature map, and the channel number of the feature map was compressed to 11ββ (β was the compression ratio, set to 4 due to performance limitations of server) to improve the computational efficiency of attention map estimation. Then, the number of channels was restored through a 3 × 3 convolutional kernel, and the attention map was reconstructed based on the effective features. This operation can be represented as:
  (6) $$\begin{array}{c} f_{in}^{CATM}=F_{cat}\left(f_{in}^{TIR},f_{in}^{VIS}\right)\\ f_{PA1}^{CATM}=\sigma \left(W_{PA1}*f_{in}^{CATM}+B_{PA1}\right)\\ f_{PA2}^{CATM}=\delta \left(W_{PA2}*f_{PA1}^{CATM}+B_{PA2}\right) \end{array}$$
  where $F_{cat}\left(\cdot \right)$ represents the concatenation operation along the channel dimension. $\delta \left(x\right)={\left(1+e^{-x}\right)}^{-1}$ is the Sigmoid function, which is used to restrict the range of the output attention map values to (0,1), ensuring that no error occured during testing and training. Meanwhile, we apply the feature sub-module to the input tensor $f_{in}^{CATM}$, and obtain the feature map $f_{feat}^{CATM}$ that stores texture information. This operation can be represented as:
  (7) $$f_{feat}^{CATM}=\sigma \left(W_{feat}*f_{in}^{CATM}+B_{feat}\right)$$
  Finally, the feature map $f_{feat}^{CATM}$ and attention map $f_{PA2}^{CATM}$ were multiplied point by point, and added to the infrared feature map $f_{in}^{TIR}$ to introduce the structural features of visible light images and obtain the updated infrared features $f_{out}^{TIR}$. This operation can be represented by the following formula:
  (8) $$f_{out}^{CATM}=f_{in}^{TIR}+f_{PA2}^{CATM}\cdot f_{feat}^{TIR}$$
- **Global Residual Connection and Hierarchical Feature Fusion:** In this task, there is a strong correlation between input features and the output image. Shallow features typically retain a significant amount of low-frequency information. Additionally, as the network goes deeper, an optimization challenge called gradient vanishing occurs. Global residual connection serves as a simple yet effective solution for addressing these issues. It enables the network to concentrate on reconstructing the image’s high-frequency information, reduces resource waste, and simultaneously resolves the gradient vanishing problem. However, relying solely on global residual connections during the network inference process cannot fully utilize the abundant image features generated, resulting in information redundancy. As the network depth increases, the spatial representation capacity gradually decreases, while the semantic representation capacity increases. Therefore, fully exploiting these features can enhance the quality of the reconstructed image. To address this issue, we adopted a hierarchical feature fusion mechanism that sent the output of each CATM to the endpoint before upsampling for processing. Considering the significant amount of redundancy in these features, we added a feature fusion layer, which acts as a bottleneck layer to selectively extract relevant information from the hierarchical features. This layer is crucial for improving network efficiency and performance. The operation can be represented by the following formula:
  (9) $$f_{c}=f_{b,n}^{TIR}+\left[W_{c}*F_{cat}\left(f_{out}^{CATM,1},f_{out}^{CATM,2},\cdots ,f_{out}^{CATM,M}\right)+B_{c}\right]$$
  where $f_{out}^{CATM,m}\left(m=1,2,\cdots ,M\right)$ represents the output of the m-th CATM in the reconstructed network, *M* represents the total number of CATMs. $f_{b,n}^{TIR}$ represents the input infrared feature map of the super-resolution reconstruction network for the n-th stage.
- **Upsamle Module (UM):** Upsampling methods have been extensively studied in super-resolution networks. Some studies process feature maps at low resolutions, and then directly upsample and reconstruct the features to the target scale, which can reduce some computational cost. However, these methods are not conducive to achieving high magnification ratios and convenient interaction of multimodal information. Our proposed network gradually performs feature extraction and information fusion while the feature map is being constantly upsampled by a factor of 2 in each stage, in order to introduce rich texture details of visible light images at different scales. The feature $f_{c}$ was input into UM and upsampled by 2× through bilinear interpolation. Then, the updated features were filtered using a 3 × 3 convolution kernel to reduce the block effect in the feature maps. This process can be formalized as follows:
  (10) $$f_{o}^{}=W_{u}*F_{up↑}\left(f_{c}\right)+B_{u}$$
  where $F_{up↑}$ represents the operation of bilinear interpolation.

#### 3.1.3. High-Frequency Detail Fusion Subnetwork

In order to maximize the utilization of visible light image information, we specially set up a high-frequency detail fusion network to further refine the infrared reconstruction images at the target scale. As it is difficult to control the computational complexity and spatial complexity of the network when operating on HR images, which is not conducive to training and inference, we designed a simple network structure consisting of three pairs of convolutional layers, three CATMs, and one reconstruction layer. The specific structure is shown in Figure 2b.

### 3.2. Loss Function

**Figure 5:**
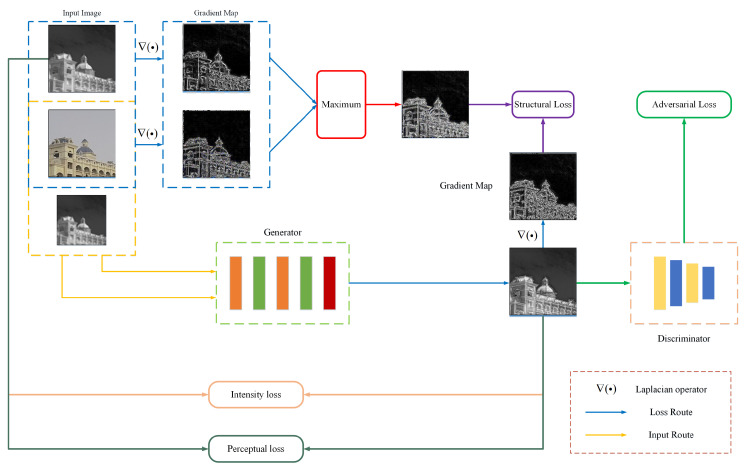
Training Process of the Neural Network. The architecture of the generator is shown in Figure 2. In order to achieve better supervision, we adopted a Markovian discriminator (PatchGAN) [40] as the discriminator to preserve high-resolution details.

To train the network proposed in this study, it was necessary to measure the similarity between the network output and Ground Truth(GT) of the infrared image, and restore the thermal information as much as possible. At the start of the third section, we emphasized the need to recover not only the known details in the infrared image, but also texture features that had been lost due to the imaging mechanism with the assistance of visible light images. Therefore, we designed a hybrid loss function, including intensity loss, structure loss, adversarial loss, and perceptual loss, to ensure the real thermal information while retaining valuable multimodal feature. The training process of neural network is shown in Figure 5.

The intensity loss is designed to retain low-frequency information of infrared images, and the main schemes include L1 and L2 loss functions. Many studies have shown that the L1 loss function is superior to the L2 loss function in terms of optimizability and reconstruction quality [24,39], so we adopted the L1 loss as the intensity loss. For the given input training samples {x,y,z}, in which *x*, *y*, and *z* are, respectively, the LR versions of infrared images, visible light images (Ref) as the reference image, and the HR version of infrared images. The intensity loss can be represented by the following formula:

(11) $$L_{i}\left(\theta \right)=\frac{1}{N}\sum_{n=1}^{N}\left|G(x,y|\theta )-z\right|$$

where G(·,·) represents the proposed network model in this article, and θ represents the weight parameters of the network model.

The role of the structural loss is to guide the network in obtaining sufficient complementary features from visible light images, which will result in the preservation of high-frequency details of both infrared and visible light images in the reconstructed image. We proposed a gradient-based structural loss to train the network to acquire this ability, employing salient features present in the infrared and reference images as a training target. The following equation represents the loss:

(12) $$L_{s}\left(\theta \right)=\frac{1}{N}\sum_{n=1}^{N}\left|\left|\nabla G(x,y|\theta )\right|-\max\left(\left|\nabla y\right|,\left|\nabla z\right|\right)\right|$$

where *∇* represents the gradient operator; we used the Laplace operator. Although the utilization of structural loss has the benefit of preserving rich high-frequency details, the ablation study conducted in Section 4.3 indicated that its implementation may lead to serious image distortion. This ultimately results in inaccurate thermal information, especially at the edges and texture details of the image. To address this issue, we added both adversarial loss and perceptual loss into the hybrid loss. These constraints facilitated the generation process of the network and ensured that thermal information in the image was preserved. Furthermore, these additions improved the overall quality of the reconstructed image. Specifically, adversarial and perceptual losses can be represented as follows:

(13) $$\begin{array}{c} L_{adv}\left(\theta \right)=\frac{1}{N}\sum_{i=1}^{N}{∥1-D(G\left(x_{i},y_{i}|\theta \right))∥}^{2}\\ L_{p}\left(\theta \right)=\frac{1}{N}\sum_{i=1}^{N}\sum_{j\in \Omega }{∥\varphi_{j}\left(G\left(x_{i},y_{i}|\theta \right)\right)-\varphi_{j}\left(z_{i}\right)∥}^{2} \end{array}$$

where D(·) represents the discriminator network and $\varphi_{j}\left(\cdot \right)$ represents the feature map of *j*-th layer in the VGG19 network. The hybrid loss we proposed can be represented by the following equation:

(14) $$L_{total}\left(\theta \right)=L_{i}\left(\theta \right)+L_{s}\left(\theta \right)+\lambda L_{adv}\left(\theta \right)+L_{p}\left(\theta \right)$$

where λ is the weight factor of the adversarial loss, which is used to balance the magnitude of other loss function values and adversarial loss. It was set to 0.1 based on experimental settings. Our ultimate goal was to minimize the value of the hybrid loss and obtain the corresponding network parameter weights, as shown in the following equation:

(15) $$\hat{\theta }=\arg\min_{\theta }L_{total}\left(\theta \right)$$

### 3.3. Training Strategies

Although introducing the information of visible light images in reconstruction image can greatly enrich the texture details in the reconstructed image, high-quality visible light images cannot always be obtained under all conditions, often being affected by conditions such as lighting and smoke. In practical application scenarios, infrared images have the characteristics of all-weather and strong anti-interference abilities; the imaging quality is also more stable. Therefore, we hoped that low-resolution infrared images could be used as the main information source in the reconstruction process, with visible light information as supplementary information. To improve the robustness of the proposed method, based on the network structure and loss function we designed, a modal switching training strategy was proposed.

During each single training epoch, we initially input multimodal data of infrared and visible light images to train all weight parameters in the network, which enabled the network to learn the ability to obtain information from input infrared images and visible light images as reference, and reconstruct high-quality images. Subsequently, to prevent significant performance deterioration of the network when no reference images are present, we input infrared images and a black image (filled with zeros) to remove the input visible light images used as references. In this process, only those structure of the network associated with infrared images were updated during training and inference, which enabled the network to attain capabilities similar to single image super-resolution. Therefore, while the reference input was being removeded, we temporarily froze all CATM that were involved in fusion parts of high frequency details and super-resolution reconstruction, as well as the encoder used to process visible light features. During this process, we set their convolutional kernels and biases to zero for a temporary period, so that no updates would be made to these parameters and thereby not impact the infrared branch’s training. Finally, The loss function could be trained as normal, without modification in this stage, as the structural loss adropts the maximum value strategy to introduce visible light information.

By using this method, we trained the network proposed by them to reconstruct high-resolution infrared images while reducing reliance on reference images in subsequent trials. During a single round of training, the discriminator was updated only once to prevent mode collapse.

## 4. Experiment

### 4.1. Experimental Environment and Dataset Settings

Our proposed network was trained and on a hardware environment with an Intel (R) Core (TM) i9-13900KF CPU, 64.0 GB of RAM and a NVIDIA GeForce RTX 4090 GPU. We used the PyCharm 2021.3.2 software platform on the Windows 11 operating system, alongside the PyTorch 1.10.1 deep learning framework. The training process took 44.3 h overall, while for each image, the testing speed was 0.62 s.

**Figure 6:**
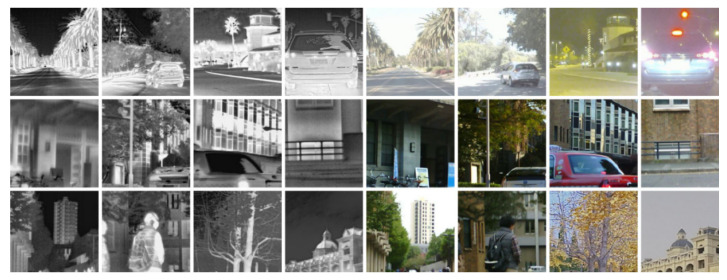
Visualization of samples from the trainging dataset.

Deep learning, as a data-driven technology, necessitates a significant amount of well-registered thermal infrared-visible light images for training data. To achieve this objective, we combined three popular multimodal datasets: M3FD [41], FLIR ADAS, and TISR [42]. Sample images from the dataset are exemplified in Figure 6. We partitioned the dataset into three sets, namely, training set, testing set and validation set with the ratio of 8:1:1. This step was conducted to evaluate the generalization capability of our proposed algorithm. Additionally, we trained and tested all other comparative methods using the same dataset.

The dataset consisted of a total of 1394 infrared and visible light images of complex scenes, including urban, road, and forest environments, with all images completed at the pixel-level alignment. To achieve data augmentation, all images in the training set were flipped and rotated, and then cropped into image blocks with a size of 256 × 256. Furthermore, we simulated degradation by downsampling the infrared images via bicubic interpolation to obtain the corresponding LR input images.

We trained our model using the ADAM optimizer and set $\beta_{1}=0.9$, $\beta_{2}=0.999$, and $\varepsilon =10^{-8}$. We set the minibatch size to 16, initial learning rate to 5 × 10^−4^, and trained the model for a total of 200 epochs. We reduced the learning rate to 0.1 at the 100-th and 150-th epochs.

### 4.2. Comparative Experiments

**Figure 7:**
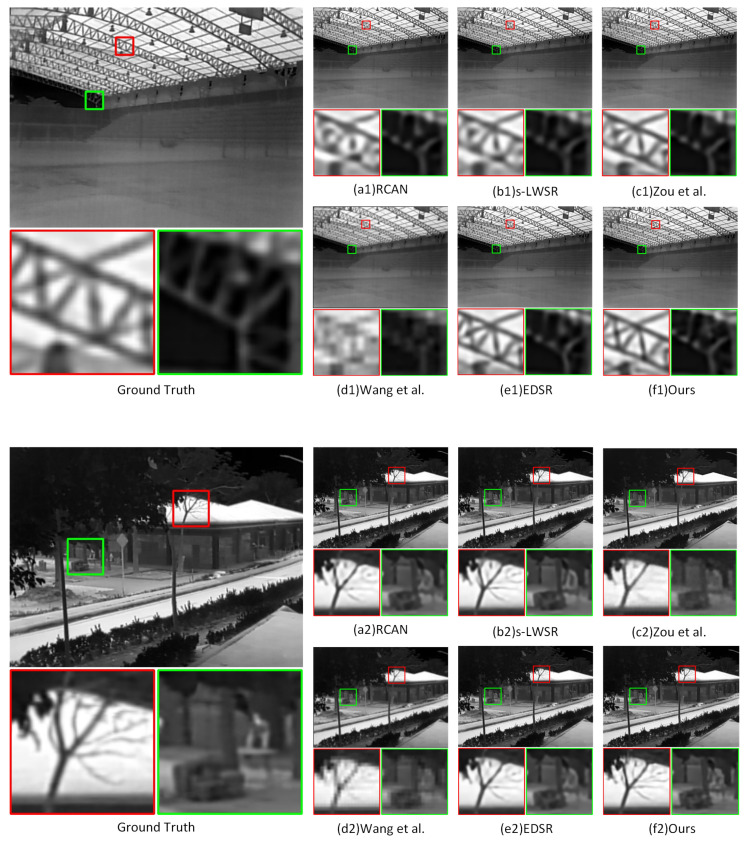
Comparison of SISR results of thermal infrared images under multimodal fusion using different methods. Zoom in for best view.

In order to demonstrate the effectiveness and superiority of our proposed method, we conducted comparative experiments on multiple classical or state-of-the-art (SOTA) methods in the same test environment. Firstly, we removed the visible light images and information conversion mechanism in the network to test the ability of our proposed method to perform single image super-resolution (SISR) without reference image guidance. In this experiment, we compared RCAN [22], EDSR [16], s-LWSR64 [19], Zou et al. [43] and Wang et al. [37]’s methods. The qualitative analysis, as shown in Figure 7, demonstrates the infrared super-resolution reconstruction results (4×) of three scenarios. Meanwhile, we present the quantitative analysis results of each method on the 8× and 4× test datasets in Table 1, mainly using the peak signal-to-noise ratio (PSNR) and the structural similarity index (SSIM) as the metrics.

In the SISR at the 4× scale, our proposed method performs comparably to EDSR in terms of performance and outperforms all other comparison methods, with slightly lower PSNR but better SSIM. It is worth noting that EDSR, as a rather large model, has about 43M parameters, while our network has only around 700 K trainable parameters (excluding frozen weight parameters), with significant advantages in both computational efficiency and memory usage. At 8× super-resolution reconstruction, our proposed method outperforms other methods, and is more suitable for high-resolution reconstruction than other methods. In terms of visual imaging performance, our proposed method effectively restores the original low-frequency information in infrared images, and is more prominent in reconstructing texture details. Wang et al.’s method, compared to the method proposed in this paper, shows a serious degradation of image quality both in objective metrics and visual perception after masking the reference image, and this is because their training strategy and information transmission mechanism cannot adapt to this situation, while our method effectively avoids this problem.

Overall, in the task of SISR, without introducing external information, the restoration performance of using only single image super-resolution methods to restore high-frequency information was limited, which may be affected by the amount of data and the difficulty of the task. From another perspective, the experiment also verifies that in the super-resolution reconstruction of multimodal information fusion, it is feasible to achieve high-quality single image super-resolution without reference images by using a modal switching strategy for training.

**Figure 8:**
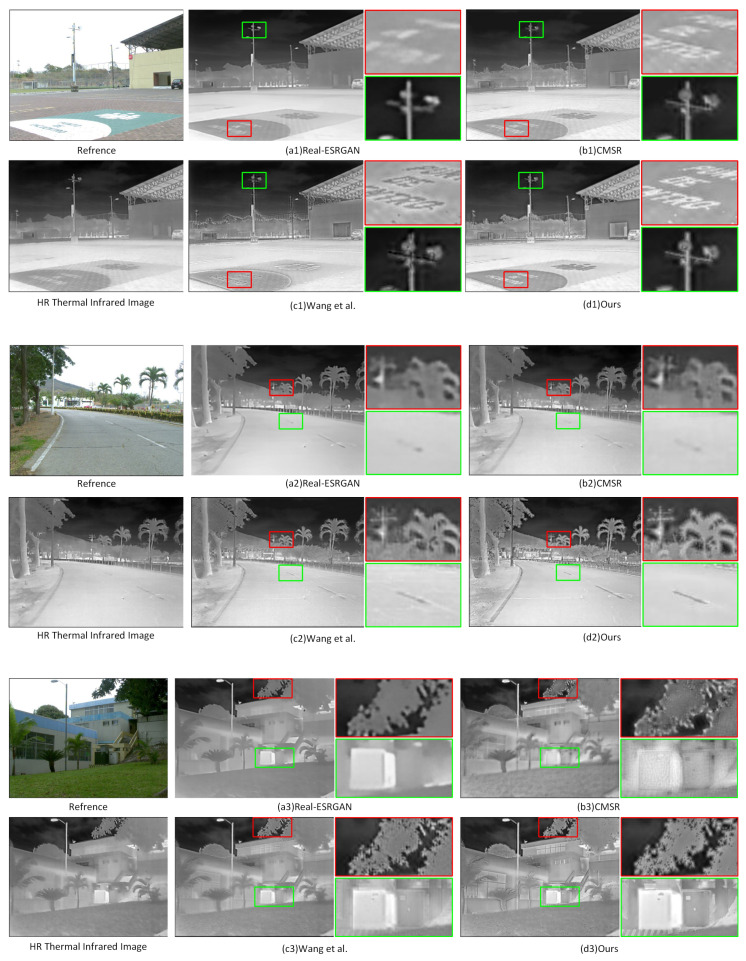
Comparison of multimodal SR results of thermal infrared images under multimodal fusion using different methods. Zoom in for best view.

After verifying the infrared super-resolution reconstruction ability of the network, we studied the effect of image super-resolution through multimodal fusion with a reference image. As discussed in Section 3, unlike SISR tasks, we no longer considered the original high-resolution infrared image as the Ground Truth, but rather aimed to restore ideal and high-quality infrared images using multimodal sensor fusion. We selected Real-ESRGAN [27], CMSR [44], and Wang et al. [37] as comparative methods to consider the network’s ability to enhance the details of infrared super-resolution reconstructed images with a reference input. The qualitative analysis is shown in Figure 8. From a visual perspective, our method not only obtained clear, high-contrast, and detail-rich infrared images but also avoided generating false textures. There were no visible artifacts or blurs compared to other contrast methods, which benefited from the neural network’s feature extraction and information transmission capabilities. To further verify, we used a reference index to analyze the correlation between the reconstructed image and thermal information (i.e., the intensity of the infrared image), including Peak Signal-to-Noise Ratio (PSNR), Structural Similarity (SSIM), Learning-based Image Perceptual Similarity (LPIPS) [45], and Mutual Information (MI). To compare the image quality generated by different methods, we also added non-reference evaluation metrics to evaluate the enhanced-detail infrared images, including Entropy (EN), Average Gradient (AG), Edge Intensity (EI), and Spatial Frequency (SF). The quantitative comparison results are shown in Table 2, where the best and second-best values for each indicator are marked in red and blue, respectively.

In general, our proposed method outperformed other reference-based comparison methods, which indicates that our images have richer details, better contrast, and preserve more infrared thermal information. Although Real-ESRGAN is superior to our algorithm in reference-based metrics, this is due to the fact that our algorithm introduces more additional information to reasonably predict some high-frequency details that are not present in the original infrared image, which would result in a certain degree of decline in reference-based metrics. However, the actual image quality can be significantly improved. The result is consistent with the qualitative analysis results of generated image quality, fully demonstrating the effectiveness of our proposed method.

### 4.3. Ablation Study

Our approach has been proven superior through the comparative experiments we conducted. Subsequently, to determine the effectiveness of our proposed improvements, we conducted a series of ablation studies.

#### 4.3.1. Ablation Studies of Network Structure

**Figure 9:**
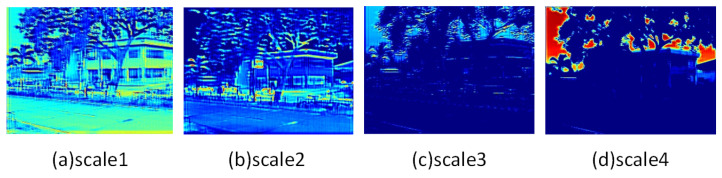
Visualization of feature maps at different scales in the HDDM.

Firstly, we investigated the impact of three mechanisms, Multi-Scale (MS), Information Distillation (ID), and CEAM, in the primary unit HDDM of the FEM on the network reconstruction performance. We observed their performance changes in the SISR task to test their ability to extract features and reconstruct images from infrared images. Table 3 shows the quantitative results. It can be seen that the main improvements, including multi-scale branch, feature distillation, and channel attention, significantly improved the network performance, with all metrics showing improvement. We display the feature maps of different scales in our multi-scale module in Figure 9. It can be seen that this structure can adaptively divide the features into different-frequency components and extract them. Our structure has achieved a good balance between performance and efficiency and can efficiently and effectively extract information from input images.

We replaced the self-attention module with three different modes: point-wise addition, channel attention, and spatial attention. We conducted experiments to evaluate their impact on performance. This primarily evaluated the performance of visible light image information when generating images using different information fusion mechanisms. The quantitative results are presented in Table 4. The attention mechanism outperforms the point-wise addition calculation mode, as evidenced by the results, which proves the importance of the learnability of information transmission. Compared to the other two attention mechanisms, our proposed CATM generated images with finer details, and had an overall better quality, which was supported by several performance metrics. This validates the effectiveness and rationale of the proposed CATM, which has the ability to extract more relevant information from the reference image.

#### 4.3.2. Ablation Study of Loss Function

**Figure 10:**
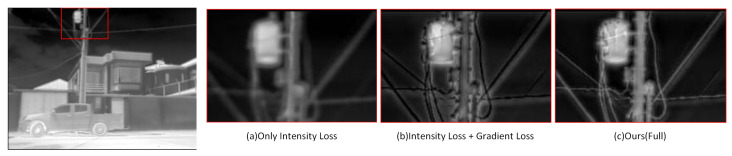
Comparison of reconstruction results using different loss functions. Zoom in for best view.

We conducted ablation experiments to analyze the composition of the loss function and to evaluate the effect of different combinations of loss functions on the quality of reconstructed images. The focus of our research was on gradient loss and adversarial loss as they are the primary approaches for achieving image reconstruction and detail enhancement. We compared the reconstruction effects of the network under three conditions, including only pixel loss, with gradient loss, and complete hyibrid loss. Figure 10 and Table 5 display the specific subjective effects and indicators discerned. After adding the loss functions, the image quality was significantly improved subjectively, with improved details and enhanced contrast and sharpness. The reference metrics showed a significant decrease with the addition of gradient and adversarial loss while the non-reference metrics displayed a significant improvement.

The purpose of using reference metrics is to measure the difference between the generated images and the GT. However, the thermal images as GT are limited by the imaging mechanism and affected by various factors, resulting in changes to the original signal, such as blurring or noise interference. Therefore, it is necessary to comprehensively judge the reconstruction ability of the network through non-reference metrics and qualitative analysis results. Obviously, the network trained with perfect hybrid loss has the best image reconstruction quality. In contrast, although the non-reference metrics have improved without introducing adversarial loss, a lot of infrared thermal information has been lost. The addition of adversarial loss can effectively solve this problem because the discriminator can prompt the generator to learn the implicit infrared image features. The lack of gradient loss makes it difficult to obtain enough texture details from the reference image, resulting in blurred reconstructed images. Therefore, our proposed hybrid loss can effectively restore the infrared thermal information in the image and obtain enough features from the reference image to enhance the texture details in the SR image.

#### 4.3.3. Ablation Study of Training Strategy

**Figure 11:**
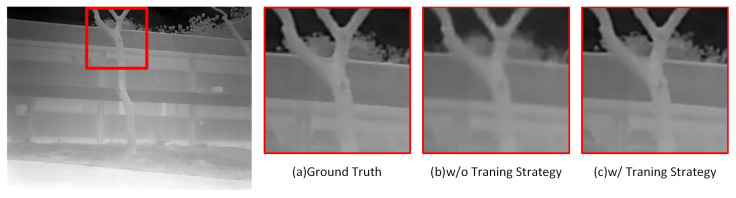
Comparison of reconstruction results of w/ and w/o training strategy.

Finally, we examined the effectiveness and necessity of the proposed training strategy through experiments. Figure 11 and Table 6, respectively, show the qualitative and quantitative analysis results of using and not using this training strategy in image reconstruction. Under SISR, if this learning strategy is not used, the quality of the reconstructed infrared images is poor, after masking the reference image and the corresponding network structure. This is mainly because the reconstruction process overly relies on the image information in visible light images. Although some of this information exists in infrared images, ineffective constraints during the training process still lead to serious network performance degradation, as demonstrated by the performance of Wang et al.’s method in the SISR comparative experiment. The network trained using this training strategy performs well in the SISR task and can reconstruct high-quality images without relying on visible light images. In the reference super-resolution task, both methods show quite similar reconstruction quality and achieve good performance, indicating that parallel training or inference of SISR and reference super-resolution tasks is feasible.

This experiment proves that the training strategy proposed in this paper can effectively optimize the network, enabling it to maximize the use of effective information in the input infrared image. It is possible to rely solely on the infrared image for reconstruction in situations where visible light reference images are missing or of poor quality, which improves the robustness of our method and provides more options for practical applications.

## 5. Conclusions

In this paper, we proposed a thermal infrared image super-resolution reconstruction method based on multimodal sensor fusion, which included a multimodal super-resolution reconstruction network, a novel hybrid loss function, and a corresponding training strategy. Our multimodal super-resolution reconstruction network adopted an iterative super-resolution approach to gradually incorporate visible light features of different scales, which could better adapt to large-scale thermal infrared image super-resolution. We designed a hierarchical expansion distillation module to extract features from thermal infrared and visible light images, which was lightweight and high-performance, contributing to generating better reconstruction results. Additionally, we proposed a cross-modal information transformation module with pixel-level attention to achieve more efficient and accurate information fusion between the two modalities. To reasonably supplement lost texture details, a hybrid loss function is proposed, which could fuse and enhance salient details in different modalities while maintaining correct thermal information, improving the imaging quality of generated images. Moreover, we proposed a training strategy for multimodal sensor fusion super-resolution to reduce the network performance degradation caused by missing or low-quality reference images, improve the network’s robustness and expand the scope of application in practical scenarios. Through extensive experimentation and comparison with various state-of-the-art methods, our method has demonstrated good performance in both visual quality and quantitative metrics, and improved the reconstruction quality of the images to some extent, validating the potential of our method.

## Figures and Tables

**Table 1:** Benchmark test results for SISR.

Methods	Prameters	4×		8×	
		PNSR	SSIM	PNSR	SSIM
RCAN	16 M	31.66	0.8724	28.20	0.7107
s-LWSR64	2.27 M	31.67	0.8894	28.18	0.7098
Zou et al.	3.73 M	31.84	0.8863	28.40	0.7121
EDSR	43.09 M	32.21	0.8913	28.38	0.7166
Wang et al.	573.6 K	21.33	0.6042	-	-
Ours	698.2 K	32.15	0.8921	28.41	0.7283

**Table 2:** Benchmark test results for multimodal SR.

Methods	Ref.	PSNR	SSIM	LPIPS	MI	EN	AG	EI	SF
Origin TIR	-	-	-	-	-	7.1152	2.3342	30.6074	14.1549
Real-ESRGAN	×	30.23	0.7130	0.1728	3.0812	7.1295	1.6568	20.8512	6.3397
CMSR	✓	29.52	0.6623	0.2213	1.7308	6.8232	3.4218	32.1385	12.2585
Wang et al.	✓	29.38	0.6570	0.2513	1.7603	6.9754	3.0916	37.8797	13.0632
Ours	✓	30.04	0.7041	0.1869	2.8672	7.6473	4.2393	49.9074	18.9905

**Table 3:** Results of ablation study on the composition of HDDM structure.

MS	ID	CEAM	Param	PSNR	SSIM
✓	×	×	14.5 K	31.84	0.6997
✓	✓	×	14.5 K	32.02	0.7002
✓	✓	✓	15.4 K	32.15	0.7041

**Table 4:** Results of ablation experiments on information transformation methods.

Transformation Type	PSNR	SSIM	EN	AG	EI	SF
point-wise add	23.2113	0.6377	4.6732	2.9369	21.1218	9.5865
Channel Attention	25.8466	0.6902	6.6181	3.6187	31.7487	12.3742
Spatial Attention	28.7214	0.6911	7.2657	4.1258	42.0281	15.8656
Ours	30.0418	0.7041	7.6473	4.2393	49.9074	18.9905

**Table 5:** Benchmark test results for multimodal SR of thermal infrared images.

$L_{i}\left(\theta \right)$	$L_{s}\left(\theta \right)$	$L_{\mathrm{adv}}\left(\theta \right)+L_{p}\left(\theta \right)$	PSNR	SSIM	EN	AG	EI	SF
✓	×	×	33.6149	0.9012	7.0281	2.2627	30.6074	14.1833
✓	✓	×	28.8282	0.6702	7.6657	4.4251	52.0372	16.7221
✓	✓	✓	30.0418	0.7041	7.2657	4.1258	42.0281	15.8656

**Table 6:** Results of ablation study on the training strategy.

Training Strategy	SISR		Multimodal SR			
	PSNR	SSIM	EN	AG	EI	SF
×	25.64	0.6130	7.1545	4.2258	40.8564	14.9313
✓	32.15	0.7041	7.2657	4.1258	42.0281	15.8656

## Data Availability

Not applicable.
